# Expression of oncofetal antigen glypican-3 associates significantly with poor prognosis in HBV-related hepatocellular carcinoma

**DOI:** 10.18632/oncotarget.9892

**Published:** 2016-06-07

**Authors:** Li Wang, Liuhong Pan, Min Yao, Yin Cai, Zhizhen Dong, Dengfu Yao

**Affiliations:** ^1^ Research Center of Clinical Medicine, Affiliated Hospital of Nantong University, Nantong 226001, China; ^2^ Department of Medical Informatics, Medical School of Nantong University, Nantong 226001, China; ^3^ Department of Immunology, Medical School of Nantong University, Nantong 226001, China; ^4^ Department of Diagnostics, Affiliated Hospital of Nantong University, Nantong 226001, China

**Keywords:** hepatocellular carcinoma, glypican-3, prognosis, tissue microarrays, immunohistochemistry

## Abstract

Hepatocellular carcinoma (HCC) is one of the most common malignancies worldwide with poor prognosis. However, its prognostic evaluation is still an urgent problem. The objectives of this present study were to investigate oncofetal antigen glypican-3 (GPC-3) expression in HCC and their match para-cancerous tissues by the array technology with immunohistochemistry and estimate its value as a novel prognostic marker for HCC. The incidence of GPC-3 expression was 95.7 % in the cancerous tissues with significantly higher (*χ*^2^ = 33.824, *P* < 0.001) than that in the para-cancerous tissues (52.2 %). Abnormal expression of GPC-3 in HCC tissues was markedly related to poor or moderate differentiation (*P* < 0.001), hepatitis B virus (HBV) infection (*P* = 0.004), periportal cancer embolus (*P* = 0.043), and tumor-node- metastasis staging (*P = 0.038*). According to the univariate and multivariate analysis, the overall survival of HCC patients with high GPC-3 level was significantly worse than those with low or without GPC-3 expression (*P* < 0.001), suggesting that abnormal GPC-3 expression should be an independent prognostic factor for HBV-related HCC patient's survival.

## INTRODUCTION

Hepatocellular carcinoma (HCC) is one of the most common and fatal malignancies worldwide with very poor prognosis [1–3], which is associated with a background of chronic persistent infection of hepatitis B virus (HBV) or hepatitis C virus (HCV) [4]. Their infections along with alcohol and aflatoxin B_1_ intake are widely recognized as etiological agents in HCC [5]. Most of HCC patients died quickly because of the rapid progression, hepatic resection or transplantation is the only potential treatment for HCC [6], and the options are rather limited [7], with its insensitive to radiotherapy or chemotherapy, higher rate of recurrence after surgery, and metastasis, leading to a poor prognosis [8]. Therefore, improving the early diagnosis and looking for an effective treatment become an urgent problem [9]. Although alpha-fetoprotein (AFP) is a useful marker for HCC diagnosis, the false- negative AFP concentration alone may be as high as 40% at early stage of HCC or remain normal in 15 ~ 30% of advanced patients. New markers such as hepatoma specific gamma-glutamyl transferase (HS-GGT) [10] and hepatoma-specific AFP (HS-AFP) [11] have been developed to improve the diagnostic sensitivity and specificity for HCC patients. However, the overall results for HCC prognosis have been unsatisfactory [12].

Glypican (GPC) is a family of heparan sulfate proteoglycans that are bound to the cell surface by a lipid anchor. Six members (GPC1~6) of this family have been identified in mammals. GPC-3 is located at Xq26.1 area on chromosome, which is bound to the cell surface through a glycosylphosphatidylinositol anchor [13]. New finding of recent research, GPC-3 expression is closely associated with hepatocyte malignant transformation [14] and is a specific oncofetal biomarker for HCC diagnosis [15, 16]. GPC-3 as an oncofetal antigen was in favors of cell proliferation and metastasis [17, 18] and could be an emerging molecular target for HCC gene therapy. However, the value of hepatic GPC-3 as a prognostic biomarker for HCC remains to be clarified [19]. The objectives of this present study were to investigate the hepatic GPC-3 expression and cellular distribution in HCC and their matched-surrounding tissues by the array technology with immunohistochemistry and analyze the clinical value of GPC-3 as a novel molecular marker for HBV-related HCC patients' prognosis.

## RESULTS

### GPC-3 expression in HCC tissues

**Figure 1:**
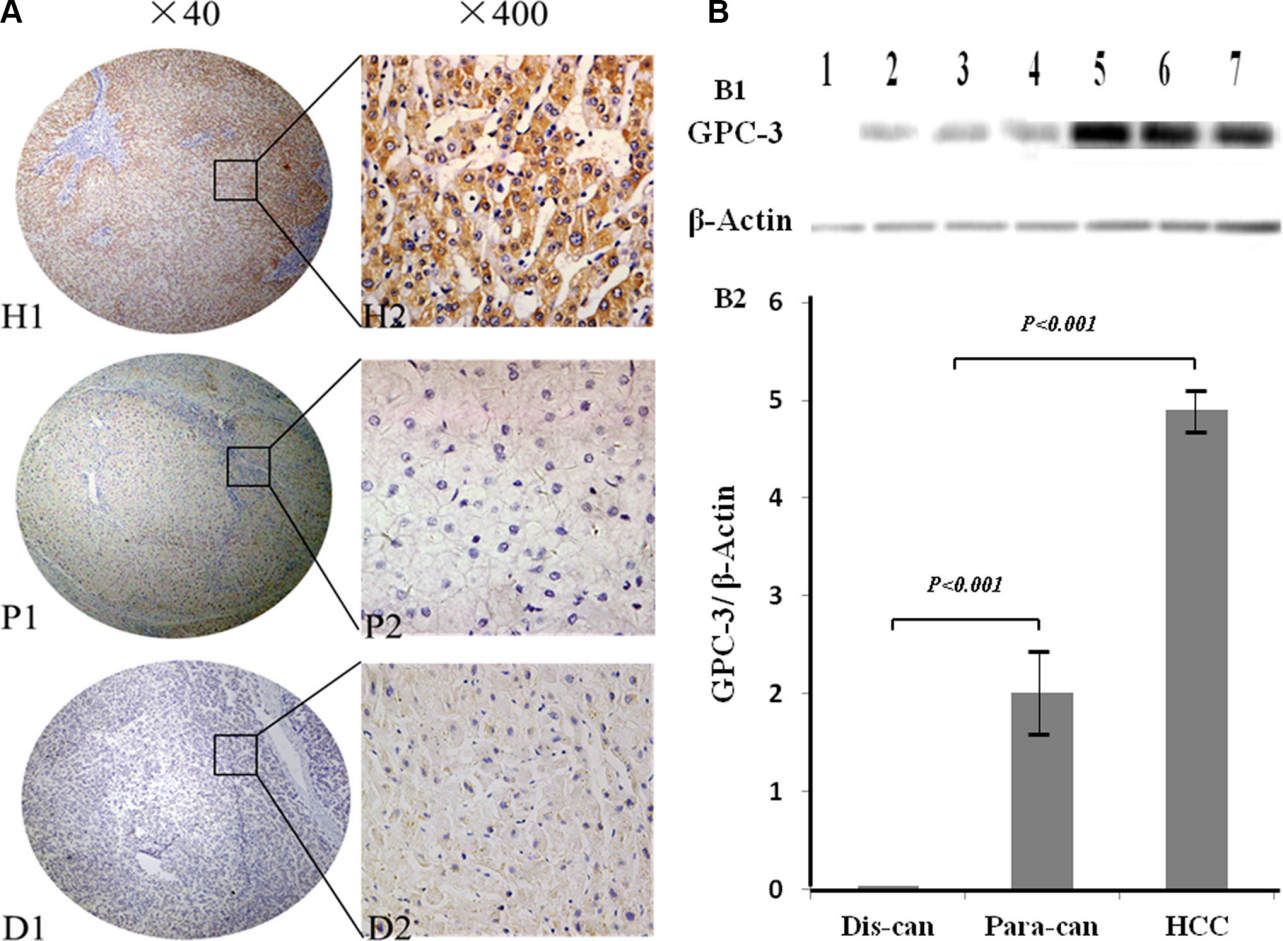
GPC-3 expression and cellular distribution between HCC and noncancerous tissues (**A**) the expression of hepatic GPC-3 was analyzed on tissue microarrays by immunohistochemistry with the primary mouse anti-human GPC-3 antibodies. The positive GPC-3 expression with brown staining particles was distributed in the cytosol or membrane of hepatocytes or only a few cell nuclei. H1 and H2, the stronger straining of GPC-3 expression in the HCC tissues; P1 and P2, the light straining of GPC-3 expression in the matched surrounding tissues; and D1 and D2, the distal cancerous tissues without any positive staining. H1, P1 and D1 original magnification × 40, and H2, P2 and D2 original magnification × 400; (**B**) the analysis of hepatic GPC-3 expression by Western blotting (B1), and the ratio of hepatic GPC-3 to β-actin (B2) with *lane 1* from the distal cancerous tissues; *lane 2, 3, and 4* from the paracancerous; and *lane 5, 6, and 7* from the HCC tissues. GPC-3: glypican-3; H1, H2 or HCC, hepatocellular carcinoma tissues; P1, P2 or Para-can, para-cancerous tissues; and D1, D2 or Dis-can, distal cancerous tissues.

Hepatic GPC-3 expressions and its cellular distribution in cancerous-, their matched surrounding-, or distal cancerous tissues analyzed by tissue microarrays with immunohistochemistry using anti-human GPC-3 antibodies are shown in Figure 1. The strongest positive GPC-3 with brown staining particles was distributed in the cytoplasm and membrane of hepatocytes or only a few cell nuclei (Figure 1A H1 and 1A H2) in the cancerous tissues. The weaker positive GPC-3 with light staining was distributed in the cytoplasm of hepatocytes in their matched para-cancerous tissues (Figure 1A P1 and 1A P2). However, no positive GPC-3 staining was found in their matched distal cancerous tissues (Figure 1A D1 and 1A D2). The stronger expressions of GPC-3 expression in the HCC tissues were confirmed by the analysis of Western blotting (Figure 1B), and the relative ratio of hepatic GPC-3 to β-actin was about 5 times high in the HCC tissues and only 2 times in their surrounding tissues.

### Comparative analysis of GPC-3 expression among different tissues

The summaries of hepatic GPC-3 staining and its expressing intensity in the cancerous, their matched surrounding or distal tissues from 69 patients with HCC are shown in Table 1. The incidence of hepatic GPC-3 expression in cancerous tissues (95.7%, 66 of 69) was significantly higher than that in their surrounding tissues (52.2%, 36 of 69; χ^2^ = 33.824, *P* < 0.001) or the distal cancerous tissues (0%, 0 of 69; χ^2^ = 126.50, *P* < 0.001). The significant difference of liver GPC-3 expressing intensity was found between cancerous and their surrounding tissues (Z = 7.968, *P*< 0.001) or the distal cancerous tissues (Z = 10.815, *P*< 0.001), with higher expression (++ ~ +++) in 78.3% (54 of 69) cancerous-, lower or no expression (0 ~ +) in 88.4% (61 of 69) their surrounding-, and none expression in 0% (0 of 69) their distal cancerous tissues. Although the hepatic GPC-3 with lower or no expression (0 ~ +) in the surrounding tissues, however, the significantly different of GPC-3 incidence (χ^2^ = 48.706, *P* < 0.001) or intensity (Z = 6.899, *P* < 0.001) was found between the surrounding tissues and the distal cancerous tissues.

**Table 1:** Incidence of GPC-3 expression and its intensity in HCC tissues

Group	*n*	GPC-3		χ^2^	*P* value	GPC-3				Z	*P* value
		Neg	Pos			-	+	++	+++		
HCC	69	3	66			3	12	52	2		
Para-can	69	33	36	33.824	< 0.001*	33	28	8	0	7.968	< 0.001*
Dis-can	69	69	0	126.5	< 0.001*	69	0	0	0	10.815	< 0.001*

* Compared with the HCC group. There was the significantly different of hepatic GPC-3 incidence (χ^2^ = 48.706, *P* < 0.001) or intensity (Z = 6.899, *P* < 0.001) between the para-cancerous group and the distal cancerous group. **HCC**: hepatocellular carcinoma; **Para-can**: para-cancerous tissues; **Dis-can**: distal cancerous tissues; **Neg**., negative staining; **Pos**., positive staining; **GPC-3**: glypican-3.

### Hepatic GPC-3 expression at different staging of HCC

**Figure 2:**
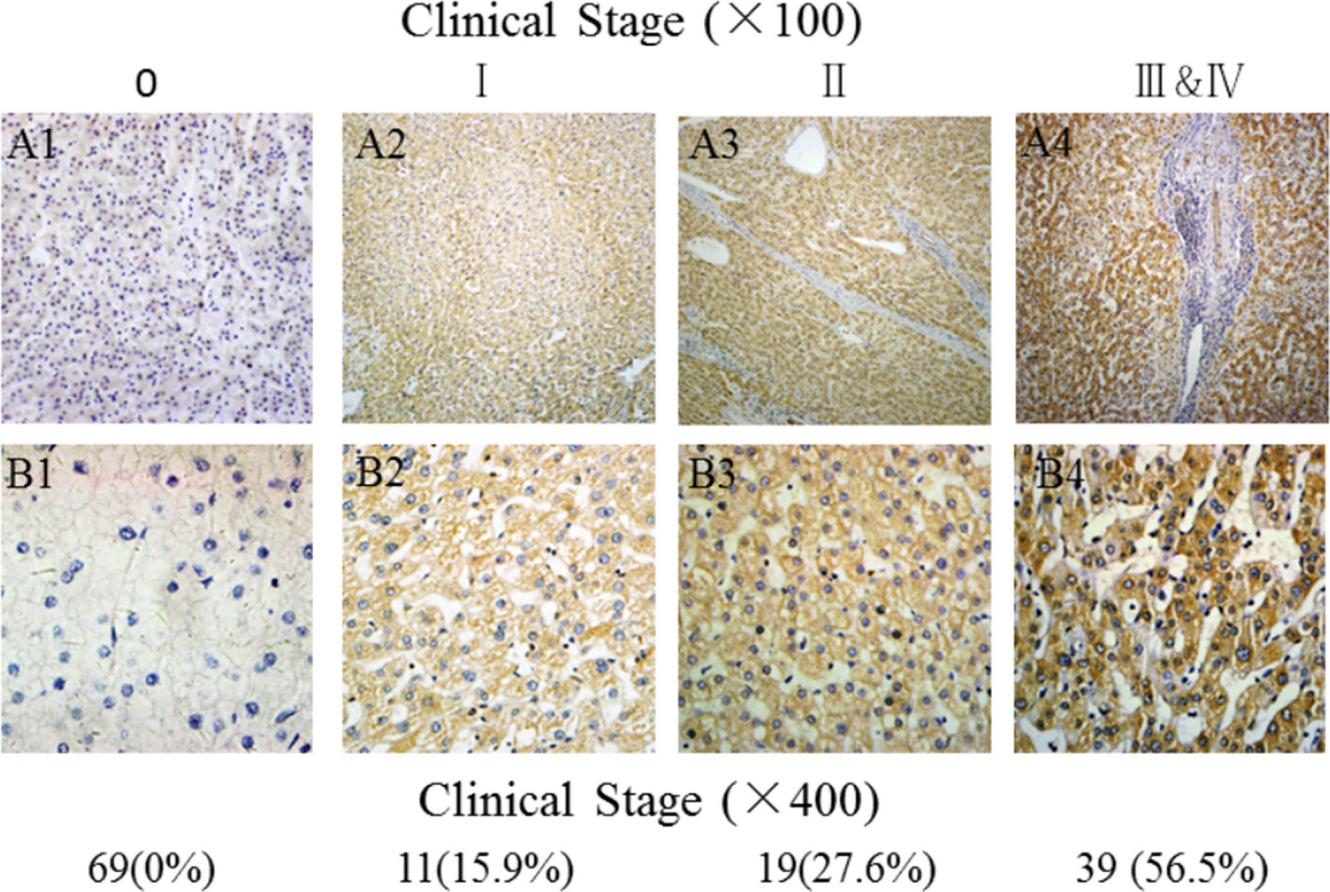
Comparative analysis of GPC-3 expression in HCC tissues at different stages A1 and B1, the low or without GPC-3 expression in the para-cancerous tissues, and A2~A4 and B2~B4, the brown staining of GPC-3 expression with gradually increasing from stage I, II to III~IV of HCC tissues; Original magnification × 100 from Figure 2 A1 to Figure 2 A4 or × 400 from Figure 2 B1 to Figure 2 B4); **HCC**, hepatocellular carcinoma; **GPC-3:** glypican-3.

The relationship between hepatic GPC-3 expression and clinical staging of HCC is shown in Figure 2. According to the IUAC clinical staging criteria of HCC patients, there was 11 cases at staging I (15.9%, 11 of 69), 19 at II (27.6%, 19 of 69), and 39 at III & IV (56.5%, 39 of 69) among total 69 cancerous tissues. The incidences of high or low GPC-3 expression in HCC tissues were 45.5% (5 of 11) or 54.5% (6 of 11) at I staging, 52.6 % (10 of 19) or 47.4% (9 of 19) at II staging, 100% (39 of 39) or 0% (0 of 39) at III & IV staging, respectively. The brown GPC-3 expressions were gradually increasing in different staging with very strength staining at advanced stage.

### Clinicopathologic characteristics of GPC-3 expression

The clinicopathologic features of hepatic GPC-3 expression in HCC tissues are shown in Table 2. The levels of GPC-3 expression in the cancerous tissues were associated with poor ~ moderate differentiated grade (*P* < 0.001), HBV infection (*P* = 0.004), and periportal cancer embolus (*P* = 0.043). No significant associations were founded between GPC-3 expression and gender, age, liver cirrhosis, tumor node metastasis (*P* = 0.038), tumor number or AFP level.

**Table 2:** Clinicopathological characteristics of GPC-3 expression in HCC tissues

Groups	*N* of patients (% of total)		GPC-3 expression, *n* (% of total)		χ^2^	*P* value
**Differentiation**					16.476	**< 0.001**
Poor~ Moderate	64	92.75	63	91.30		
Well	5	7.25	3	60.00		
**Tumor number**					0.871	0.151
Single	54	78.26	51	94.44		
Multiple	15	21.74	15	100.00		
**Liver cirrhosis**					1.406	0.236
Absent	24	34.78	22	91.67		
Present	45	65.22	44	97.78		
**HBV infection**					8.254	**0.004**
Absent	19	27.54	16	84.21		
Present	50	72.46	50	100.00		
**Periportal embolus**					4.077	**0.043**
Absent	30	43.48	27	90.00		
Present	39	56.52	39	100.00		
**AFP (ng/mL)**					1.568	0.211
< 400	46	66.67	43	93.48		
≥ 400	23	33.33	23	100.00		
**TNM staging**					4.326	**0.038**
I–II	29	42.03	26	89.66		
III–IV	40	57.97	40	100.00		

**HBV:** Hepatitis B virus; **AFP:** α-fetoprotein; **TNM:** tumor node metastasis; **GPC-3:** glypican-3.

### High GPC-3 expression with shorter survival in HCC patients

**Figure 3:**
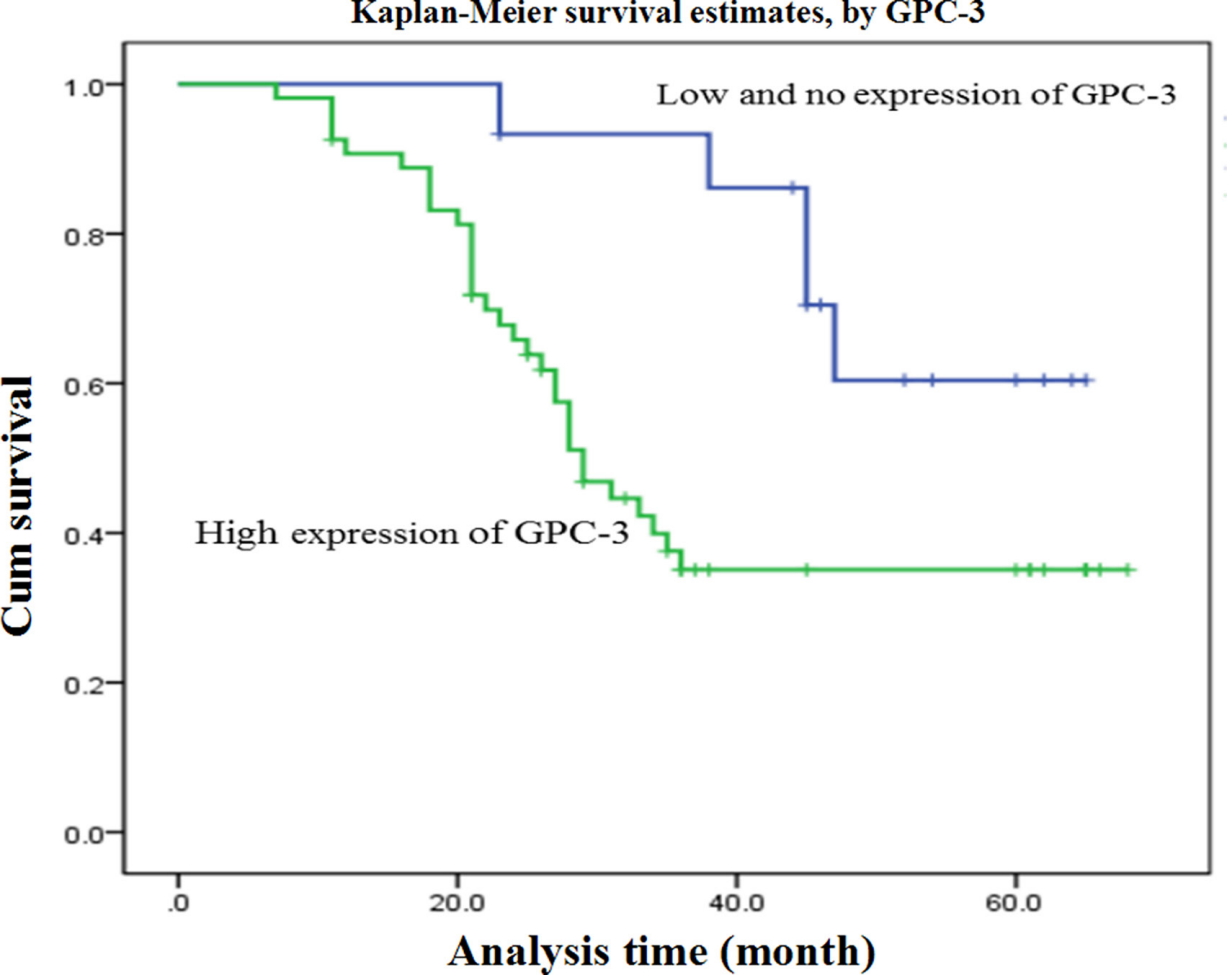
Overall survival curves of 69 patients with HCC GPC-3 expression curves were calculated according to the Kaplan-Meier method. The accumulative survival curves of 69 patients with HCC were made according to HCC tissue expressing low or higher GPC-3 level (log-rank test, *P* < 0.001). The green line is the higher GPC-3 group; and the blue line is the lower or without GPC-3 group. HCC, hepatocellular carcinoma; GPC-3: glypican-3.

As expected, the over-expression of hepatic GPC-3 showed a significant relationship with 5-year survival of 69 HCC patients (*P* < 0.001). Meanwhile, certain HCC clinical prognostic factors, such as liver cirrhosis (*P* = 0.007), HBV infection (*P* = 0.014), also showed a statistically significant correlation with 5-year survival rate based on COX regression univariate analysis (Table 3). All these factors were enrolled in a multivariable analysis. High GPC-3 expression (*P* < 0.001), liver cirrhosis (*P* = 0.008) and HBV infection (*P* = 0.006) were all identified as independent predictive factors for worse HCC outcome. The Kaplan-Meier survival curves (Figure 3) demonstrated that HCC patients with high GPC-3 expression had a significantly shorter survival time compared with those low or no GPC-3 expression.

**Table 3:** Univariate and multivariable analysis of HCC prognostic factors for 5-year survival

Variable	Univariate analysis			Multivariable analysis		
	HR	*P* > |z|	95% CI	HR	*P* > |z|	95% CI
**Differentiation**						
Low vs. high*	1.218	0.633	0.543–2.730			
**Tumor number**						
Single vs. multiple	0.905	0.853	0.316–2.597			
**Liver cirrhosis**						
Absent vs. present	3.831	0.007	1.443–10.168	3.119	0.008	1.351–7.200
**HBV infection**						
Absent vs. present	0.346	0.014	0.148–0.808	0.328	0.006	0.149–0.726
**Periportal embolus**						
Absent vs. present	5.392	0.089	0.775–37.502			
**AFP (ng/mL)**						
< 400 vs. ≥ 400	0.768	0.240	0.495–1.193			
**TNM staging**						
I–II vs. III–IV	0.243	0.120	0.041–1.446			
GPC-3 expression*						
Low vs. high	12.697	< 0.001	3.097–52.050	4.259	< 0.001	2.030–8.934

* As shown in Table 2, the high GPC-3 expression (++~+++) group; and the no or low GPC-3 expression (− ~ +) group. **HBV:** Hepatitis B virus; **AFP:** α-fetoprotein; **TNM:** tumor node metastasis; **GPC-3:** glypican-3; **HR:** Haz ratio; **CI:** confidence interval.

## DISCUSSION

HCC is one of the most common malignancies worldwide, but treatment outcomes have remained generally poor [1, 2]. In China, most of HCC cases are associated with HBV infection [20, 21]. Activation of some key signal molecules in inflammation-related liver diseases regulates the expression of inflammation response- or cancer related genes [22, 23]. GPC-3 gene is located in upstream of the Wnt signal pathway that is involved in the initiation, generation, and tumor development, and up-regulated in HBV-related liver malignancies [19]. It promotes the growth of HCC by stimulating canonical Wnt signaling because the structural requirements for GPC3 activity are cell type specific, and its core protein is processed by a furin-like convertase, In this study, the expression and cellular distribution of hepatic GPC-3 in HCC were investigated to assess its prognostic value of HCC.

Application of imaging and molecular genetics technologies have elucidated lots of HCC characteristics that is a multi-step process from precancerous lesions progress into early or progressed HCC, and the close surveillance and treatment of these lesions will be useful to improve the survival rates of HCC patients [24]. GPC-3 isn't detected in normal livers, sera of HBV-related benign liver diseases or healthy populations [12]. Increasing oncofetal GPC-3 expression was detected during the malignant transformation of rat hepatocytes, with brown granule-like staining localized in tumor parts of atypical hyperplasia [14]. There were strong brown staining particles in the cytoplasm and membrane or a few nuclei with high GPC-3/β-actin ratio in HCC, light staining in the para-cancerous-, and none in the non-cancerous tissues. The significantly different of GPC-3 incidence or intensity was found between HCC and their surrounding- or distal cancerous-tissues, indicating that GPC-3 expression could associate significantly with HCC progression [19].

Clinical studies have reported that GPC-3 is a valuable specific marker for HCC diagnosis [25, 26]. In this study, the relationship between GPC-3 expression and clinical HCC staging was investigated. According to the IUAC clinical staging criteria of HCC patients, the high GPC-3 expression were about 50% at I or II staging, and all at III & IV staging with gradually increasing and strength staining at advanced stage. The clinicopathologic features of GPC-3 expression in HCC patients were associated with poor ~ moderate differentiated grade, HBV infection, and periportal cancer embolus. A statistically significant correlation with 5-year survival rate based on COX regression univariate analysis, more interesting is GPC-3 level and HBV infection identified as independent predictive factors for worse HCC outcome, that is, HCC patients with higher GPC-3 level, and shorter survival time compared with those lower or no expression, suggesting that GPC-3 not only be a specific biomarker but also be an independent prognostic factor for HCC.

In conclusion, the up-regulation of oncofetal antigen GPC-3 regulates the malignant transformation of hepatocytes, promotes cell proliferation, and effects on the survival rate of HCC patients through its downstream canonical or noncanonical Wnt pathway that should provide new mechanism insight into molecular-targeted therapy for HCC [27, 28] because of contributing to HCC growth and related to HBV infection. Molecular targeted therapy offers an effective option for non-surgical HCC management, but it remains be a challenge due to a lack of specific targets [29, 30]. Perhaps GPC-3 is specifically expressed in HCC but not in benign liver diseases, silencing its gene transcription by specific miRNA or anti-GPC-3 antibody could effectively inhibit HCC proliferation via the Wnt/β-catenin pathway [31]. Further work should be explored combination of miRNA plus multi-targeting strategies for HCC therapy [32].

## MATERIALS AND METHODS

### Tissues and follow-up data

A total of 69 formalin-fixed, paraffin-embedded cancerous-, 69 matched psracancerous- (more than 3 cm to cancer focus) and 69 distal cancerous-tissues (more than 5 cm) specimens [33] after surgical operation were obtained from Affiliated Hospital of Nantong University between Jan 2006 and May 2008. The patient cohort inclusion and exclusion criteria included accurate pathologic diagnosis of primary HCC, complete pathologic and follow-up data from operation to death date or last follow-up until Oct 2013. Overall survival was evaluated from surgery to death. Diagnosis was based on the criteria of the Chinese Society of Liver Cancer and Chinese Anti-Cancer Association [34]. Differentiation degree or tumor staging was according to the Edmondson grading system or the 6th edition of the tumor-node-metastasis (TNM) classification of the International Union against Cancer (IUAC). Studies were approved by the Hospital Ethics Committee, and all patients signed informed consent.

### Tissue microarrays

The tumors were dissected and pathological examination was performed with H&E. All HCC tissues were reviewed by two histopathologists. Representative areas free from necrotic and hemorrhagic materials were marked in paraffin blocks. Two mm tissue cores were taken from each representative cancerous- and their surrounding- or distal cancerous-tissue to construct the tissue microarrays (TMA) slides.

### Immunohistochemistry

TMA slides used for immunohistochemistry (IHC) were deparaffinized, and peroxidase was quenched with methanol and 3% H_2_O_2_ for 15 min. For antigen retrieval, the sections were boiled under pressure in citrate buffer (pH 6.0) for 3 min, and then incubated for 120 min with primary mouse anti-human GPC-3 antibody (ab129381; Abcam, UK) at 1:200 dilution, washing with phosphate buffered saline (PBS), incubated with horse reddish peroxidase (HRP)-conjugated goat anti-mouse IgG (A21010; Abcam, UK) for 15 min at 1:5000 dilution, and washed again with phosphate buffer solution (PBS). In finally, the slides were re-incubated with diaminobenzidine and counterstained with hematoxylin solution, dehydrated in ethanol, cleared in xylene, and cover-slipped. The negative control included empty control with normal mouse IgG instead of primary antibodies or with second antibodies only. The negative control included 0.01 mol/L PBS instead of primary, second antibodies, and immunohistochemical reagents.

### Evaluation of immunohistochemical findings

The results of IHC staining were assessed by two independent pathologists without knowledge of the clinicopathologic features. The percentages or positive intensities were classified into 4 grades: 0 or negative for none, 1 for 1~33% or weaker positive (+), 2 for 34~66% or moderately positive (++), and 3 for 67 ~ 100 % or strongest staining (+++). The scores were used as the percentage and intensity of GPC-3 as described earlier [33], and the staining intensities were defined as low (0 ~ +) or high expression (++ ~ +++).

### Extraction of liver proteins

Fifty milligrams of liver tissue was homogenized in 1 ml of PBS for 15 min at 4°C, stored at 4°C overnight, and centrifuged at 12 000 g for 15 min. The supernatant was transferred to a new 1.5 ml Eppendorf tube for analysis of total protein concentration with TBA assay.

### Western blotting

Total proteins in the supernatants were subjected to Western blotting as described previously^21^. Briefly, the protein (50 μg/lane) for each sample was separated using 10% sodium dodecyl sulfate-polyacrylamide gel electrophoresis (SDS-PAGE) and then transferred onto a polyvinylidene fluoride membrane (Beyotime, China) and blocked in 5% nonfat dry milk (Guangming, China) in Tris- buffered saline (pH 7.5, 100 mM NaCl, 50 mM Tris, and 0.1% Tween-20, TBST), incubated with anti-human GPC-3 antibodies (ab129381, Abcam, UK) overnight at 4°C, along with β-actin (New England Bio Labs, MA), and followed with horseradish peroxidase conjugated IgG (Everest Biotech, UK). The membranes were then incubated with a secondary antibody (goat anti-rabbit; New England Bio Labs) in TBST also for 1 h at room temperature. The immuno-complexes were visualized using an ECL kit (Amersham Pharmacia Biotech, UK) and images were developed with the Molecular ImagerR Gel DocTMXR System (Bio-Rad laboratories, Inc., USA).

### Statistical analysis

Data were presented as mean ± standard deviation (M ± SD) and subjected to one-way analysis of variance. Differences between groups were compared using a Student's *t* test or χ^2^ test. Kaplan-Meier analysis was to determine the significance of the main outcome and performed using SPSS20.0 software and GraphPad Prism version 5.01. A *P* < 0.05 value was set for significance.

## References

[1] Bruix J, Gores GJ, Mazzaferro V (2014). Hepatocellular carcinoma: clinical frontiers and perspectives. Gut.

[2] El-Serag HB (2011). Hepatocellular carcinoma. N Engl J Med.

[3] Jemal A, Bray F, Center MM, Ferlay J, Ward E, Forman D (2011). Global cancer statistics. CA Cancer J Clin.

[4] El-Serag HB (2012). Epidemiology of viral hepatitis and hepatocellular carcinoma. Gastroenterology.

[5] Asim M, Sarma MP, Thayumanavan L, Kar P (2011). Role of aflatoxin B1 as a risk for primary liver cancer in north Indian population. Clin Biochem.

[6] Maluccio M, Covey A (2012). Recent progress in understanding, diagnosing, and treating hepatocellular carcinoma. CA Cancer J Clin.

[7] Akoad ME, Pomfret EA (2015). Surgical resection and liver transplantation for hepato- cellular carcinoma. Clin Liver Dis.

[8] Kalyan A, Nimeiri H, Kulik L (2015). Systemic therapy of hepatocellular carcinoma: current and promising. Clin Liver Dis.

[9] Forner A, Llovet JM, Bruix J (2012). Hepatocellular carcinoma. Lancet.

[10] Yao D, Jiang D, Huang Z, Lu J, Tao Q, Yu Z, Meng X (2000). Abnormal expression of hepatoma specific gamma-glutamyl transferase and alteration of gamma-glutamyl transferase gene methylation status in patients with hepatocellular carcinoma. Cancer.

[11] Wu W, Yao DF, Yuan YM, Fan JW, Lu XF, Li XH, Qiu LW, Zong L, Wu XH (2006). Combined serum hepatoma-specific alpha-fetoprotein and circulating alpha- fetoprotein-mRNA in diagnosis of hepatocellular carcinoma. Hepatobiliary Pancreat Dis Int.

[12] Wang L, Yao M, Dong Z, Zhang Y, Yao D (2014). Circulating specific biomarkers in diagnosis of hepatocellular carcinoma and its metastasis monitoring. Tumour Biol.

[13] Kwack MH, Choi BY, Sung YK (2006). Cellular changes resulting from forced expression of glypican-3 in hepatocellular carcinoma cells. Mol Cells.

[14] Yao M, Yao DF, Bian YZ, Zhang CG, Qiu LW, Wu W, Sai WL, Yang JL, Zhang HJ (2011). Oncofetal antigen glypican-3 as a promising early diagnostic marker for hepatocellular carcinoma. Hepatobiliary Pancreat Dis Int.

[15] Li B, Liu H, Shang HW, Li P, Li N, Ding HG (2013). Diagnostic value of glypican-3 in alpha fetoprotein negative hepatocellular carcinoma patients. Afr Health Sci.

[16] Yao M, Yao DF, Bian YZ, Wu W, Yan XD, Yu DD, Qiu LW, Yang JL, Zhang HJ, Sai WL, Chen J (2013). Values of circulating GPC-3 mRNA and alpha-fetoprotein in detecting patients with hepatocellular carcinoma. Hepatobiliary Pancreat Dis Int.

[17] Yu D, Dong Z, Yao M, Wu W, Yan M, Yan X, Qiu L, Chen J, Sai W, Yao D (2013). Targeted glypican-3 gene transcription inhibited the proliferation of human hepatoma cells by specific short hairpin RNA. Tumour Biol.

[18] Yao M, Wang L, Shi Y, Qian Q, Yu D, Shi Y, Lu S, Yao D (2014). Intervention of glypican-3 genetic transcription on anti-proliferative effect of hepatoma cells with high metastatic potentiality. Zhonghua Yi Xue Za Zhi.

[19] Yao M, Wang L, Dong Z, Qian Q, Shi Y, Yu D, Wang S, Zheng W, Yao D (2014). Glypican-3 as an emerging molecular target for hepatocellular carcinoma gene therapy. Tumour Biol.

[20] Yao M, Gu X, Wang L, Cai Y, Wu W, Shi Y, Dong ZZ, Yao DF (2016). Abnormal expression of circulating nuclear factor-κB in hepatocellular carcinoma and reversal of multidrug resistance through intervening its gene transcription. Zhonghua Yi Xue Za Zhi.

[21] Sai W, Wang L, Zheng W, Yang J, Pan L, Cai Y, Qiu L, Zhang H, Wu W, Yao D (2015). Abnormal expression of Golgi protein 73 in clinical values and their role in HBV- related hepatocellular carcinoma diagnosis and prognosis. Hepat Mon.

[22] Chung W, Kim M, de la Monte S, Longato L, Carlson R, Slagle BL, Dong X, Wands JR (2016). Activation of signal transduction pathways during hepatic oncogenesis. Cancer Lett.

[23] Teng CF, Hsieh WC, Wu HC, Lin YJ, Tsai HW, Huang W, Su IJ (2015). Hepatitis B virus Pre-S2 mutant induces aerobic glycolysis through mammalian target of rapamycin signal cascade. PLoS One.

[24] Niu ZS, Niu XJ, Wang WH, Zhao J (2016). Latest developments in precancerous lesions of hepatocellular carcinoma. World J Gastroenterol.

[25] Yao M, Pan LH, Yao DF (2015). Glypican-3 as a specific biomarker for hepatocellular carcinoma. Hepatobiliary Pancreat Dis Int.

[26] Wang L, Yao M, Pan LH, Qian Q, Yao DF (2015). Glypican-3 is a biomarker and a therapeutic target of hepatocellular carcinoma. Hepatobiliary Pancreat Dis Int.

[27] Dong Z, Yao M, Wang L, Yang J, Yao D (2014). Down-regulating glypican-3 expression: molecular-targeted therapy for hepatocellular carcinoma. Mini Rev Med Chem.

[28] Allegretta M, Filmus J (2011). Therapeutic potential of targeting glypican-3 in hepato- cellular carcinoma. Anticancer Agents Med Chem.

[29] Cao H, Phan H, Yang LX (2012). Improved chemotherapy for hepatocellular carcinoma. Anticancer Res.

[30] Yao M, Wang L, Qiu L, Qian Q, Yao D (2015). Encouraging microRNA-based therapeutic strategies for hepatocellular carcinoma. Anticancer Agents Med Chem.

[31] Feng M, Ho M (2014). Glypican-3 antibodies: a new therapeutic target for liver cancer. FEBS Lett.

[32] Broderick JA, Zamore PD (2011). MicroRNA therapeutics. Gene therapy.

[33] Zhang HJ, Yao DF, Yao M, Huang H, Wu W, Yan MJ, Yan XD, Chen J (2012). Expression characteristics and diagnostic value of annexin A2 in hepatocellular carcinoma. World J Gastroenterol.

[34] Chinese Society of Liver Cancer, Chinese Anti-cancer Association (2001). Criteria of diagnosis and stage for primary liver cancer. Chin J Hepatol.

